# Determinants of willingness to share personal genomic data: a systematic review focused on health literacy

**DOI:** 10.1186/s12910-026-01456-w

**Published:** 2026-04-16

**Authors:** Marleen Schmeiss, Renate Schramek

**Affiliations:** Department of Health Sciences, Bochum University of Applied Sciences, Bochum, Germany

**Keywords:** Genomic Data Sharing, Health Literacy, Data Governance, Patient Perspectives

## Abstract

**Background:**

Genomic medicine increasingly depends on patients’ willingness to share genomic and medical data. While data sharing supports advances in personalised care, it also raises ethical and social concerns related to privacy, trust and participation. Understanding these factors requires attention to patients’ health literacy and their capacity to interpret and act upon genomic information.

**Methods:**

A systematic review was conducted according to PRISMA guidelines to identify empirical studies published between 2015 and 2025 that explored patients’ understanding of genomic information and their willingness to share data. Searches were performed in PubMed, Web of Science and Scopus. Eligible studies included qualitative, quantitative and mixed-methods designs. Findings were synthesised thematically and Nutbeam’s model of health literacy was used in the discussion to interpret the results.

**Results:**

Fifteen studies met the inclusion criteria. Participants demonstrated basic understanding of genetic terms but limited knowledge of data infrastructures and governance. Trust was a central factor influencing willingness to share data, often compensating for limited genomic literacy. Moral and altruistic motives encouraged engagement, whereas financial considerations played a minor, context-dependent role.

**Conclusions:**

Data sharing in genomic medicine relies on more than factual knowledge. Strengthening health literacy through transparent, dialogue-based, and participatory approaches can promote informed, autonomous, and ethically responsible participation in genomic research.

**Supplementary Information:**

The online version contains supplementary material available at 10.1186/s12910-026-01456-w.

## Background

Genomic medicine is an expanding field that integrates genomic data into clinical practice to enhance diagnosis, predict disease outcomes and guide individualized treatment decisions. It overlaps with concepts such as personalized and precision medicine, aiming to translate genomic insights into improved patient care [6, 15]. Databases play a central role in this process, as they enable the collection and comparison of genomic data, supporting both research and clinical precision [7, 10]. Currently, patients with rare diseases and cancer are at the center forefront of genomic medicine, as sequencing technologies facilitate more accurate diagnoses and individualized treatment approaches [3].

As genomic medicine continues to advance, its success increasingly relies on patients´ willingness to share their genomic and medical data [30]. Data sharing supports large-scale research and the development of evidence-based clinical applications. Yet, it also raises complex ethical, social and psychological questions concerning privacy, trust, and individual autonomy [1, 12]. Understanding the factors that influence patients’ willingness to share genomic data is therefore essential for fostering participation and ensuring equitable, ethically sound implementation of genomic medicine [13]. This endeavour requires a perspective that extends beyond clinical efficacy and considers how patients’ access, interpret and act upon health-related information. Decisions about genomic data sharing are not purely informational but involve evaluating privacy risks, trust in institutions and perceived social value of research [9]. Individuals must interpret complex information, weigh uncertainties and align these considerations with personal values. Health literacy therefore directly influences how people translate information into autonomous decisions about whether to share their data.

To date, existing research on genomic medicine has predominantly focused on clinical implementation, technical infrastructures and ethical principles, while comparatively little attention has been given to how patients understand genomic data sharing and the governance of genomic databases. This limited insight into patients’ perspectives on data use and storage constrains the development of educational and participatory approaches aimed at strengthening informed consent processes, improving patient-facing communication and supporting public engagement in genomic research, thereby promoting informed decision-making and fostering trust in genomic research.

One relevant lens for investigating these factors is provided by the health sciences, particularly through approaches to health literacy, such as the conceptual framework developed by Nutbeam [17]*.* His staged model of functional, interactive and critical health literacy offers a valuable perspective for understanding how patients engage with genomic information, manage uncertainty, and participate in decision-making processes. In the context of genomic medicine, this review focuses on patients’ understanding of how genomic databases operate and what it means to share genomic information within broader systems of governance and trust, rather than on the clinical application of genomic testing itself. From this perspective, data sharing constitutes a complex social practice that requires not only basic comprehension but also communicative and critical reflection on privacy, autonomy and the social value of scientific collaboration.

Therefore, this systematic review examines patients’ willingness to share medical data within genomic research and databases, alongside their understanding of genomic information, and synthesizes how these aspects have been addressed in empirical studies. The findings are discussed in relation to Nutbeam’s [17] staged model of health literacy. This framework illustrates how functional, interactive and critical forms of understanding shape perceptions of data protection, trust and motivation in the context of genomic data sharing, database participation, and engagement in research within genomic medicine.

## Research design and objectives

The research project adopts a systematic review design and is methodologically guided by the PRISMA guidelines [19]. The aim of the review is to capture the current state of knowledge about regarding patients´ understanding of the sharing of their medical, and particularly genomic, data, as well as the factors influencing their willingness to share such data in research and healthcare databases. It also examines how aspects of health literacy are addressed in research and how they shape the decision to share data [17]. Given the thematic breadth and methodological diversity of the included studies, which encompass qualitative and quantitative primary studies, the systematic review provides insight into the current state of knowledge, offering a comprehensive understanding of patients´ attitudes, motives and concerns, as well as the factors ultimately influencing their decisions.

### Search strategy

The systematic literature search was conducted in the PubMed, Web of Science, and Scopus databases. The search strings combined terms related to health literacy/patient education and genomic medicine/precision medicine, with a focus on human studies. Detailed search strings for all databases included in this review are provided in Appendix 1. Publications classified as reviews, systematic reviews, or meta-analyses were excluded. The search strategy was deliberately broad to ensure comprehensive coverage of relevant literature. As the additional combination of search terms related to data sharing would have significantly reduced the number of hits and risked omitting relevant studies, these terms were not directly integrated into the search strings. Instead, aspects of data sharing and consent for the use of genomic information were identified and coded during title/abstract and full-text screening.

### Conceptual framework: adapted PICO scheme for systematic reviews

Based on the University of Sydney's guidelines for qualitative systematic reviews, the adapted PICO framework was applied to structure the search and analysis process. PICo refers not to interventions but to key qualitative dimensions:

**Table:** 

P (Population)	patients or individuals involved in genomic medicine
I (Phenomenon of Interest)	understanding, attitudes, and willingness to share medical and genomic data
Co (Context)	clinical and research settings in which genomic data are collected, shared, or stored

As the review process includes studies employing both qualitative and quantitative research approaches, both methodologies are considered and interpreted within the applied PICO framework [29].

### Inclusion and exclusion criteria

**Fig. 1:**
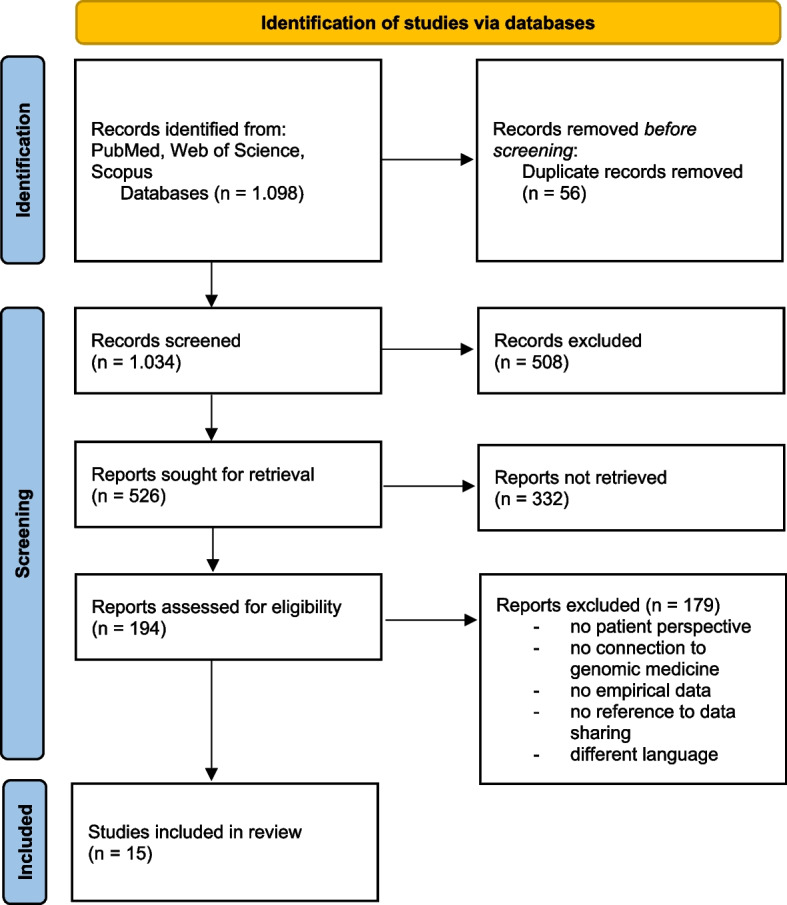
Study selection process according to PRISMA guidelines

Inclusion and exclusion criteria were established to ensure the relevance and comparability of the included studies. Studies investigating patients' understanding, attitudes, or willingness to share medical, and particularly genomic, data were included. A prerequisite was a connection to genomic medicine, for example, in the context of genetic or genomic testing procedures, sequencing programs, or biomedical databases. Only studies published between 2015 and 2025 were considered, in order to reflect the current state of knowledge in clinical and digital genomic medicine. Qualitative, quantitative, or mixed-methods studies were included, provided they reported empirical results. Publications were required to be in English or German. Studies were excluded if they did not include a patient perspective, focused exclusively on technical, methodological or bioinformatics issues, had no direct connection to genomic medicine or the sharing of genomic data, or were purely conceptual, ethical or policy-oriented, such as commentary or policy articles. A total of 1,077 publications were excluded (see PRISMA flowchart Fig. 1). Common reasons for exclusion included a lack of patient relevance, lack of relevance to genomic medicine, or absence of empirical data. These criteria ensured that only studies directly contributing to the understanding of genomics and willingness to share data in the context of genomic medicine were included in the synthesis. Relevant data from the included studies were extracted into a structured Excel spreadsheet documenting study characteristics, design, population, and key findings regarding health literacy and data sharing. Given the heterogeneity of study designs, the results were summarized narratively and thematically to integrate qualitative and quantitative evidence. A comprehensive overview of all included studies and their key findings is presented in Appendix 2.

## Results

A total of 15 studies published from 2018 to 2025 were included in the analysis. Most originated in the USA, with others from the UK, China, and Australia. Study designs ranged from qualitative interview studies to large-scale surveys. Study participants included patients, parents or adolescents who participated in genomic testing or genome sequencing programs. Table 1 presents five quantitative, six qualitative and four mixed-methods studies, listed in that order.

**Table 1:** Overview of included studies on patients’ understanding of genomic information and willingness to share data

Authors	Study Setting	Year	Title
Williams J. R., et al	USA	2018	Precision Medicine: Familiarity, Perceived Health Drivers, and Genetic Testing Considerations Across Health Literacy Levels in a Diverse Sample
Roberts, J. S., et al	USA	2018	Patient understanding of, satisfaction with, and perceived utility of whole-genome sequencing: findings from the MedSeq Project
Pacyna, J. E., et al	USA	2019	Should pretest genetic counselling be required for patients pursuing genomic sequencing? Results from a survey of participants in a large genomic implementation study
Naeim, A., et al	USA	2021	Electronic Video Consent to Power Precision Health Research: A Pilot Cohort Study
Biesecker, B., et al	USA	2025	Genomic sequencing in diverse and underserved pediatric populations: Parent perspectives on understanding, uncertainty, psychosocial impact, and personal utility of results
Lewis, C., et al	UK	2020	Young people's understanding, attitudes and involvement in decision-making about genome sequencing for rare diseases: A qualitative study with participants in the UK 100, 000 Genomes Project
Chen, H	China	2021	Privacy in breast cancer biobank: Chinese patients’ perceptions
Suckiel, S. A., et al	USA	2022	Perspectives of diverse Spanish- and English-speaking patients on the clinical use of polygenic risk scores
Spector-Bagdady, K., et al	USA	2022	Patient and Provider Perspectives on Enrollment in Precision Oncology Research: Qualitative Ethical Analysis
Noohi, F., et al	USA	2023	Diagnosis, treatment and disclosure: A qualitative exploration of participant challenges in a Monogenic Diabetes Registry
Sabatello, M., et al	USA	2024	Return of polygenic risk scores in research: Stakeholders’ views on the eMERGE-IV study
Ta Park, V., et al	USA	2021	Motivation to Participate in Precision Health Research and Acceptability of Texting as a Recruitment and Intervention Strategy Among Vietnamese Americans: Qualitative Study
Raj, M. et al	USA	2022	Public Deliberation Process on Patient Perspectives on Health Information Sharing: Evaluative Descriptive Study
Smit, A. K. et al	Australia	2022	Communicating Personal Melanoma Polygenic Risk Information: Participants' Experiences of Genetic Counseling in a Community-Based Study
Stallings, S. C., et al	USA	2023	Assessing patient-level knowledge of precision medicine in a community health center setting

### Aspect: understanding genomics

The reviewed studies revealed considerable heterogeneity in participants´ understanding of genomic medicine. While a basic familiarity with common genetic terminology was evident, comprehension declined markedly for more specialised concepts. For example, in the study by Williams et al. [31]*,* 71% of participants reported familiarity with the term 'DNA' and 63.9% with the term 'gene,' whereas only 17.9% recognized ‘biobanking’. Comparable findings were reported by Stallings et al. [25], where 77% of participants were familiar with the term 'DNA,' 25% with the term 'genomics,' and only 12% with the term 'biobank.' Importantly, these measures reflect self-reported familiarity rather than tested conceptual understanding and the studies did not consistently specify whether technical terms were formally defined in participant material.

Qualitative investigations by Lewis et al. [11] and Spector-Bagdady et al. [24] found similar results. Participants frequently used analogies – describing DNA, for instance, such as 'an instruction manual', 'fingerprint',—and referred to popular media such as 'Jurassic Park' and scientific documentaries, to conceptualise and explain genetic mechanisms.

The studies by Naeim et al. [14]*, *Noohi et al. [16]*,* and Raj et al. [20] further underscore the importance of educational approaches that support active learning. The study by Noohi et al. [16] revealed that participants with higher levels of functional and communicative health competence often compensate for missing clinical information independently through their own research. Interventions such as the use of video materials in Naeim et al. [14] improved participants’ perceived clarity and ease of understanding of consent information, while deliberative discussion formats in Raj et al. [20] demonstrated measurable pre- and post-session gains in knowledge about health privacy laws and public data governance.

### Aspect: privacy and data security

Privacy and data security turn out to be key factors influencing participants’ engagement in genomic research, although both understanding and concern vary considerably across studies. While most participants place high importance on data privacy, their understanding of how genomic data are stored, shared and managed is often limited.

In the study by Williams et al. [31], 79.4% of participants rated the privacy of genetic test results as important, yet only 17.9% were familiar with the concept of a biobank. This finding illustrates a pronounced gap between valuing confidentiality and understanding the underlying data infrastructures. Across studies, a similar discrepancy emerged between perceived and actual understanding. Participants in the studies by Pacyna et al. [18] and Roberts et al. [21] reported feeling well-informed and confident in their decision-making; however, 14% of participants in Roberts et al. [21] were unaware that their anonymized data were shared in an NIH^1^ database. Smit et al. [23] also noted that of 448 study participants, only three had questions about the whereabouts of the biological samples after the study. These findings indicate that limited awareness of long-term data storage and secondary data use may vary and is not always well understood.

Most participants understood data protection primarily as the safeguarding of personal and medical information rather than as a matter of autonomy or data ownership. Although only a minority voiced explicit concerns, many expressed fears of data breaches and potential misuse [22, 25, 26]. In addition, Sabatello et al. [22] identified that some participants emphasized a strong sense of data ownership and the right to decide who should have access to their genomic information. Some participants considered the loss of privacy as an unavoidable aspect of modern digital life [4], while others, particularly from ethnic minority backgrounds, including Vietnamese American participants, as well as older adults, described data sharing as a moral responsibility in which societal benefits outweighed personal risks [24, 28].

Given the heterogeneity of study designs, no definitive conclusions can be drawn regarding the association between consent to data sharing, income and health literacy. Nevertheless, the reviewed evidence consistently indicates that health literacy plays a predictive role in shaping attitudes toward data privacy. Participants with higher levels of health literacy expressed fewer privacy concerns [2] and showed greater confidence in the handling of genomic information. In contrast, participants from socioeconomically disadvantaged groups expressed stronger fears of data misuse [2], a pattern observed in Chen [4], where participants from minority backgrounds reported similar concerns despite health literacy not being directly assessed.

Similarly, participants with prior research experience appeared more willing to share their data within trustworthy governance frameworks [14, 18]. Although health literacy was not explicitly assessed in these studies, this greater willingness to share data may reflect higher levels of confidence and familiarity with research processes, which are conceptually related to interactive forms of health literacy. While most participants expressed trust in anonymisation procedures and institutional oversight, uncertainty persisted regarding the boundaries of data use, particularly with respect to commercial or governmental access. Lewis et al. [11] found broad trust in the NHS^2^ but also noted ambivalence toward private sector involvement. Naeim et al. [14] reported similar findings: despite perceived data security, privacy concerns remained the main reason for opting out. Educational interventions in the studies by Naeim et al. [14] and Raj et al. [20] improved understanding of data governance and encouraged a more critical reflection on the balance between privacy and public benefit. Both Naeim et al. [14] and Raj et al. [20] showed that educational initiatives improved understanding of data governance and encouraged a more critical reflection on the tension between data privacy and public benefit.

### Aspect: trust and institutional confidence

Trust was a central factor influencing participants’ willingness to share genomic and medical data across studies. It appeared in three main forms: I) institutional trust in health systems and research infrastructures [11, 16], II) relational trust in individual clinicians or researchers [14, 22, 31] and III) culturally grounded trust embedded in community and spiritual contexts [25, 28].

Institutional confidence was reflected in participants’ reassurance through data deidentification and established governance systems such as the NHS [11, 20]. The quantitative findings from Naeim et al. [14] confirmed that trust in science and researchers significantly predicted willingness to share data, while Williams et al. [31] reported that 77.4% of primary care participants expressed trust in healthcare professionals. Similarly, Sabatello et al. [22] found that trust constituted a prerequisite for data sharing, particularly when participants retained a sense of personal control.

Several studies [2, 18, 24] suggest that when participants showed limited understanding of genomic processes or data governance, willingness to participate appeared to be grounded more in trust in researchers or institutions than in detailed comprehension. In this sense, trust can be interpreted as an orienting resource that may enable decision-making under conditions of uncertainty. Stallings et al. [25] further emphasized that trust extended beyond professional relationships, with community figures, churches, and families serving as credible sources of genomic information.

### Aspect: altruism, social and economic dimensions of participation

Across studies, study participants' willingness to engage in genome sequencing and data sharing was primarily driven by moral, social, and personal motives, whereas economic considerations played a secondary, but context-dependent, role.

Altruism and the desire to contribute to science and the common good frequently emerged as key motivators. In the study by Naeim et al. [14], the majority of participants described participation as an opportunity to support medical progress: 94 of 101 respondents rated contributing to curing diseases as "very important," while others expressed hopes to advance science or help others. Comparable patterns were reported in Spector-Bagdady et al. [24]*, *Sabatello et al. [22], and Lewis et al. [11], where participation was portrayed as an act of hope and a moral legacy for the benefit of future generations. In addition to altruism, personal and familial relevance also guided engagement. Participants sought diagnostic clarity, emotional validation, or a sense of control over their own or their children's health [11, 16].

Cultural and social factors were also influential: Ta Park et al. [28] reported that Vietnamese-American participants were motivated by representation and culturally relevant treatments, linking participation to a sense of belonging and inclusion. Similarly, in Lewis et al. [11]*,* younger participants emphasized the empowerment associated with involvement in decision-making processes, describing this as a feeling of being "important and not just a blood source."

Economic considerations indirectly influenced participation. For a group of study participants, financial compensation was a relevant aspect of the decision to share data: in the study by Williams et al. [31], 24.6% of respondents valued payment as relevant for their decision. However, most study participants cited principles of fairness and access to medical care as relevant aspects for their decision to share data. In the study by Stallings et al. [25], 85% of participants named insurance coverage and 72% cost as important determinants for their decision. These economic factors appeared together with concerns about test results, family impact, privacy, and trust in healthcare professionals, suggesting that financial aspects were linked to broader questions of access and fairness rather than individual profit. Suckiel et al. [26] emphasized that financial and language barriers limited equitable participation. Participants also expressed ambivalence toward commercial participation: some viewed collaboration with industry positively, while others feared exploitation.

Overall, participation in genome research reflected intertwined moral, social, and structural dimensions. It was grounded in trust, perceived social value, and the pursuit of inclusion and autonomy rather than financial gain.

## Discussion

The studies included in this review are highly heterogeneous with respect to context, study design and population. As a result, the discussion does not seek to compare findings directly but to analyze them through the lens of Nutbeam’s [17] health literacy framework. This approach facilitates a nuanced understanding of how functional, interactive and critical forms of literacy shape participants’ perceptions of genomic data sharing, trust and decision-making.

### Functional health literacy

In Nutbeam’s [17] framework, functional health literacy represents the most basic level of literacy, focusing on the transmission and comprehension of factual health information that enables individuals to navigate health systems effectively. In genomic medicine, it extends beyond basic reading skills to include understanding key genetic and data-related terminology and interpreting these concepts in ways that allow meaningful engagement with genomic information.

The uneven development of functional health literacy observed across studies underscores a critical challenge. Familiarity with basic terms such as “DNA” or “gene” suggests that information about genomics reaches the public, yet the declining understanding of more specific concepts like “genomics” or “biobanking” indicates that current communication strategies remain largely transmissive [25, 31]. From *Nutbeam’s* perspective, this reflects on conveying facts without supporting comprehension constrains individuals’ ability to relate genomic knowledge to decisions about data use or research participation.

Metaphors and media references further illustrates how individuals construct meaning from fragmented information [11, 24]. While these analogies can render complex ideas more accessible, they also risk reinforcing superficial or misleading interpretations. This underscores the need for educational approaches that move beyond unidirectional information delivery and support the development of domain-specific literacy skills. Strengthening functional literacy requires educational approaches that translate technical information into knowledge, that is relevant, comprehensible and actionable.

### Interactive health literacy

According to Nutbeam [17], interactive health literacy refers to advanced cognitive and literacy skills that, together with social abilities, enable individuals to participate actively in everyday life. It involves the capacity to extract information, interpret communication and apply knowledge to changing circumstances. In the context of genomic medicine, this form of literacy is particularly relevant, as participation in data sharing and research requires not only understanding but also the ability to engage in dialogue, interpret complex information and adapt one’s decisions to new or uncertain situations. Across studies, trust appeared in three main forms: institutional trust in health systems and research infrastructures, relational trust in clinicians and researchers and culturally grounded trust in community or spiritual contexts.

Although participants showed limited understanding of how genomic data are stored and shared, many expressed strong confidence in publicly governed systems such as the NHS [11, 20]. This pattern can be interpreted through the lens of interactive health literacy. As described by Nutbeam [17], this level of literacy encompasses not only processing information but also the use of social and relational skills to navigate uncertainty. In this context, institutional trust frequently operates as a proxy for understanding: When individuals are unable to assess data infrastructures in detail, they tend to place their confidence in systems perceived as transparent, regulated, and accountable. Public institutions such as the NHS represent accountability and collective responsibility, providing reassurance that data are managed ethically. Such trust is not only institutional but also cultural. It reflects broader experiences of legitimacy and social protection in publicly funded health systems.

Trust in professionals appears to play a similar role. Quantitative findings indicate that confidence in scientists and clinicians enhances participants’ willingness to share data [14, 22, 31]. When individuals perceive experts as competent and transparent, trust reduces perceived risks and confers a sense of legitimacy upon decision-making. safe and meaningful. Social and community contexts further shape how trust is established. Several studies [25, 28] reported that participants relied on community leaders, family and church representatives as credible sources of genomic information. In these contexts, trust was not only institutional but relational, grounded in shared identity, care and belonging. Such social trust extends the notion of interactive health literacy beyond individual comprehension, emphasizing collective understanding and cultural resonance.

From a health literacy perspective, trust can be understood as a form of social competence. It facilitates participation not through technical expertise but through confidence in individuals and institutions. However, such reliance on trust also entails certain risks. When participation is grounded primarily in perceived legitimacy rather than informed understanding, individuals remain passive within data governance. This concern echoes broader debates in biobank and digital data governance, where scholars argue that long-term legitimacy cannot rely on institutional trust alone but requires participatory and transparent governance structures that actively involve publics in decision-making [5, 8].

Strengthening interactive health literacy in genomic medicine thus requires transparent, dialogue-based communication. In this context, trust becomes not merely passive reliance but an informed and active mode of participation.

### Critical health literacy

Building on Nutbeam’s model (2000), critical health literacy refers to advanced cognitive and social skills that enable individuals to critically appraise information and to apply this understanding to exert greater control over decisions and life circumstances. Within the context of genomic medicine, this includes evaluating ethical and social implications of data sharing.

Across the reviewed studies, participants’ decisions to share genomic data appeared to be guided more by moral reasoning than by technical understanding. Many described their participation as a contribution to science and the common good, often framing it as an act of hope or solidarity with future generations [14, 22, 24]. Such motives indicate an emerging form of critical literacy, in which individuals connect their personal decisions to notions of collective benefit and ethical responsibility. At the same time, participants’ reflections on fairness, representation, and access indicate a growing awareness of structural inequalities in genomic medicine. Cultural and social belonging were particularly salient among minority groups, who associated participation with visibility and inclusion [28]. Younger participants expressed a desire to be regarded as more than research resources, seeking a sense of personal meaning and recognition in their involvement [11]. Collectively, these perspectives suggest that participants engage with genomic research not merely as patients but as moral and social actors.

Economic considerations add a further dimension to the critical aspects of participation in genomic research. While a minority of participants mentioned financial incentives, most emphasised fairness, insurance coverage and affordability as key conditions for sharing their data [25, 31]. In the study by Stallings et al. [25]*,* conducted in the United States, participants’ references to insurance coverage highlight that the decision about participation were closely tied to concerns about healthcare access and cost. These findings suggest that concerns about data sharing are not limited to privacy or trust but also encompassing broader questions of access and security within different health systems. Ambivalence toward commercial involvement [27] indicates a mixed perception of industry participation in genomic research. While some participants viewed collaboration with private actors as beneficial for innovation, others expressed concern about potential exploitation or unequal access to future benefits.

Taken together, these results suggest that elements of critical health literacy are emerging within participants’ moral and social reasoning. However, this form of literacy often remains implicit, manifesting through values such as fairness, altruism, and trust rather than through explicit critical awareness of data governance. Promoting critical health literacy in genomic medicine therefore requires educational and participatory approaches that support individuals in making their implicit reflections more explicit and in linking ethical awareness with informed and autonomous decision-making about data sharing.

## Limitations

This review was conducted systematically and with attention to methodological rigour, yet some limitations must be acknowledged. The included studies were heterogeneous in design and context, which limits generalisability but allowed for an analytical synthesis across qualitative, quantitative and mixed-methods research designs. Differences in the structures of health systems and cultural contexts likely shaped the values, attitudes, trust, and perceptions of study participants, including regarding data protection, and probably also influenced their decision to participate in the study and/or their attitude toward their perceived willingness to share their own data.

Health literacy was not consistently assessed across studies; related constructs such as knowledge or perceived confidence were often used instead, reducing comparability. The search was limited to English and German publications. One Spanish-language^3^ and one inaccessible article^4^ could not be included, although their inclusion is unlikely to have altered the findings. Finally, as genomic medicine continues to evolve, the included evidence reflects a specific period (2015–2025).

## Conclusion

This systematic review investigated patients' attitudes toward sharing their own genomic data, how their understanding influences this decision, and which factors affect their willingness to share their data in genome databases and for genomic research. Using *Nutbeam's* model of health literacy *(2000)* as an analytical tool, the results show that the willingness to share personal data in genomic medicine is shaped by different levels of health literacy: functional, interactive, and critical. While basic health literacy supports the understanding of genomic information, informed and ethically sound decisions about data sharing require the ability to communicate, evaluate, and reflect on the broader implications of genomic data use.

Trust proved to be a key factor, often compensating for a limited understanding of data management. This finding underscores the importance of education and consent processes that combine trust with transparency and understanding. Promoting health literacy in genomics should therefore focus on participatory and reflective educational approaches that enable informed, autonomous, and socially responsible decisions about data sharing.

## Supplementary Information

Supplementary Material 1.

Supplementary Material 2.

## Data Availability

All data analysed during this systematic review are included in this published article and its supplementary information files. Appendix 1 contains the full search strategy and Appendix 2 provides the complete tabulated results of the included studies.

## Footnotes

1 NIH stands for National Institutes of Health, the U.S. government’s primary agency for biomedical and public health research, funded through federal appropriations.

2 NHS stands for National Health Service, which refers to the publicly funded healthcare system in the UK, primarily funded through taxes and National Insurance contributions.

3 One relevant French-language article could not be included: Desprès, C., & Mamzer, M.-F. (2022). Le consentement éclairé en question dans le cadre de collections en biobanque. Bulletin du Cancer, 109(9), 948–959. https://doi.org/10.1016/j.bulcan.2022.03.012

4 One identified article could not be accessed in full text: Watson, S., Pacyna, J. E., Olson, J. E., & Sharp, R. R. (2025). Assessing decisional regret among biobank sample donors. Biopreservation and Biobanking, 23(2), 145–151. https://doi.org/10.1089/bio.2024.0053
