# Supplementary material for: Determinants of willingness to share personal genomic data: a systematic review focused on health literacy
Source: BMC Med Ethics. 2026 Apr 16;27:87. doi: 10.1186/s12910-026-01456-w (PMC13109877; doi:10.1186/s12910-026-01456-w)
Supplement: Supplementary file 1 — Supplementary Material 1. [file 12910_2026_1456_MOESM1_ESM.docx]

**Applied search strings in PubMed, Web of Science and Scopus**

**PubMed:**

Search: ( "health literacy"[All Fields] OR "health competence"[All Fields] OR "patient knowledge"[All Fields] OR "patient competence"[All Fields] OR "health education"[MeSH Terms] OR "Patient Education as Topic"[MeSH Terms] ) AND ( "genomic medicine"[All Fields] OR "personalized medicine"[All Fields] OR "precision medicine"[All Fields] ) NOT ("review"[Publication Type] OR "systematic review"[Publication Type] OR "meta-analysis"[Publication Type]) Filters: in the last 10 years, English, German, Humans

Number of hits: 292

**Web of Science:**

TS=("health literacy" OR "health competence" OR "patient knowledge" OR "patient competence" OR "health education") AND TS=("genomic medicine" OR "personalized medicine" OR "precision medicine") AND DT=(Article) AND PY=(2015-2025)

Number of hits: 92

**Scopus:**

The literature search in Scopus was conducted using a broad search string applied to the fields title, abstract, and keywords. To address the platform’s technical limitations regarding logical operators, formal restrictions such as publication period (2015–2025), language (German and English), and document type (article) were subsequently applied using Scopus’ filter function.

("health literacy" OR "health competence" OR "patient knowledge" OR "patient competence" OR "health education")

AND ("genomic medicine" OR "personalized medicine" OR "precision medicine")

Number of hits: 714
