# Supplementary material for: Determinants of willingness to share personal genomic data: a systematic review focused on health literacy
Source: BMC Med Ethics. 2026 Apr 16;27:87. doi: 10.1186/s12910-026-01456-w (PMC13109877; doi:10.1186/s12910-026-01456-w)
Supplement: Supplementary file 2 — Supplementary Material 2. [file 12910_2026_1456_MOESM2_ESM.docx]

**Result: Overview of findings on patients’ understanding of genomics and aspects of data sharing**

| Authors; Year; Study Setting | Study Aim | Study Design/ Population | Instruments assessing genomic knowledge and literacy | Understanding genomics | Aspects of Data Sharing |
| --- | --- | --- | --- | --- | --- |
| Williams J. R., et al.; 2018; USA | To examine patients’ familiarity, perceptions, and values related to precision medicine across health literacy levels and ethnic/racial groups. | cross-sectional survey study/ n=252 Adults (≥18 years) receiving primary care | Health literacy was assessed with the 3-item Brief Health Literacy Screen (BHLS) developed by Chew et al. (2004, 2008). Items were rated on a five-point Likert scale, resulting in total scores ranging from 3 to 15. A score of 10 or higher indicated adequate health literacy (McNaughton et al., 2014).  Thirteen items assessed participants’ familiarity with precision medicine–related terms, including terminology drawn from genetics (Erby et al., 2008), personalized medicine (KRC Research, 2014), and the broader precision medicine literature.  Twelve items measured perceptions of factors influencing personal health, addressing domains such as genetics, health literacy, socioeconomic status, cultural background, and environment.  Six items evaluated values related to decisions about genetic testing, focusing on factors identified in prior research as relevant for participation in genetic testing and genetic research, including trust, privacy, cost, counseling, receipt of results, and compensation (Martinez et al., 2017; Saulsberry & Terry, 2013; Walker et al., 2014). | 23.8% were familiar with the term precision medicine.   71.0% of participants were familiar with the term DNA and 63.9% with gene, whereas only 17.9% reported familiarity with biobank.  Participants with higher health literacy were more familiar with genetic terms, but overall knowledge of modern genomic concepts was still low. | 79.4% of participants considered privacy of genetic test results important, with similar importance across ethnic and racial groups.  83.7% of participants valued receiving their test results, and 77.4% valued trust in healthcare professionals.  24.6% of participants considered receiving payment for blood or tissue donation important.  Participants with higher health literacy rated privacy and trust slightly higher than those with lower health literacy. |
| Roberts, J. S., et al.; 2018; USA | To examine patients’ experiences, understanding, satisfaction and perceived utility of whole-genome sequencing (WGS) in clinical practice. | Randomized controlled trial (RCT) comparing WGS + family health history vs. family health history alone./ 202 adult patients; mean age 55 years within the MedSeq Project from two clinical contexts: cardiology (n=102) and primary care (n=100) practices | 11-item genomic sequencing knowledge scale was used to measure genetic literacy, assessing understanding of the benefits and limitations of genomic sequencing21-item true/false quiz developed for the study was used to assess understanding of the informed consent process, covering domains such as study purpose, procedures, risks and benefits, and policies regarding the return of sequencing results | Higher genetic literacy was significantly associated with better informed consent knowledge (B = 0.337, p = 0.03), but not with decisional regret or satisfaction with communication. Suggests that genomic understanding supports informed decision-making but does not directly influence emotional outcomes. | 100% participants knew their results would be entered into their medical records14% were unaware that their de-identified genomic data would be shared with external researchers (e.g., in the NIH dbGaP database). |
| Pacyna, J. E., et al.; 2019; USA | To assess the decision-making of individuals pursuing genomic sequencing without pre-test genetic counseling, and to describe the extent to which those who declined counseling experienced decisional conflict or difficulty making a testing decision. | Survey/ n = 3,037 adults recruited from the Mayo Clinic Biobank and the Vascular Disease Biorepository | 100-item surveyBehavioral Risk Factor Surveillance System (2014)Multidimensional Health Locus of Control ScaleKnowledge scale on genomic sequencing capabilities and limitationsprior genetic testing, self-reported health status, health literacy (Chew et al., 2004)Decisional Conflict Scale (DCS; 16-item, modified for research participation context)Primary outcomes:Decisional conflict (measured with the modified DCS)Decisional latency (time required to make the decision: immediately, after hours, or after days)Additional variables: discussions with family, friends, healthcare providers or research staff during decision-making. | 95% of respondents felt they made an informed choice and 98% were satisfied with their decision to participate in genomic sequencing85%–90% reported understanding the risks and benefits80% indicated they felt knowledgeable about the specific implications most relevant to themAssociation with decisional conflict: Higher genetic knowledge (OR = 0.90; CI 0.87–0.95), higher education (OR = 0.86; CI 0.79–0.95) and previous genetic testing experience (OR = 1.42; CI 1.05–1.92) were significantly associated with lower decisional conflict (DCS < 25).Health literacy as a predictor: Participants with higher health literacy were more confident in coping with results and demonstrated reduced decisional conflict. Those with limited literacy or less self-efficacy showed greater uncertainty and delay in deciding to participate.Decisional latency: Participants who decided immediately (76%) had lower conflict (mean DCS = 14.4) than those who deliberated for hours (DCS = 20.1) or days (DCS = 24.4), showing a clear link between literacy, confidence and decisiveness. | High informed consent comprehension (95%) and willingness to participate without counseling suggest trust and acceptance of data sharing. However, prior biobank participation may limit generalizability to less research-experienced populations. |
| Naeim, A., et al.; 2021; USA | To explore patients’ acceptance and understanding of an electronic, animated video consent for donating remnant biospecimens and to examine which factors influenced their decision to opt in or opt out, such as trust, perceived usefulness and privacy concerns. | Pilot study using a quantitative survey design to evaluate video consent process/ n=173 patients (≥18 years) completed the electronic video consent | Two 4–5 minute electronic video consent formats (text-based and animated); both included NIH-informed consent elements, information about collaboration and potential genomic data useAfter watching the video: participants were asked whether they wished to opt in or opt out of donating remnant biospecimens for research and whether they agreed to be recontacted for future studiesThree five-point Likert-scale questions evaluating: usefulness; ease of understanding; trustworthiness of the consent videosAdditional survey items for opting in: to help science; family; find cures; or opting out: privacy concerns; misunderstanding; opposition to tissue use10-item questionnaire, developed by the study team based on validated instruments, measured attitudes: biomedical research; optimism; altruism; privacy; social support; justice; conflict of interest. | Participants’ decision to opt in was strongly associated with positive attitudes toward science, altruism, and trust in researchers. The belief that sharing biological samples is safe due to privacy protections (OR = 4.5, AUC = 0.88) and that research contributes to public health (OR = 5.5, AUC = 0.88) were among the strongest predictors of participation. | n = 101 opting in: n = 94 cited contributing to the cure of diseases as very importantn = 90 hoped to advance sciencen = 84 wanted research to help family, friends or others in the futuren = 20 participants opting out: n = 18 reported privacy concerns as their main reasonn = 5 did not want their biological samples used for othersn = 2 did not understand the consent process |
| Biesecker, B., et al.; 2025; USA | To examine parents’ perceived understanding, personal utility, and test-related distress after receiving their child’s genome-scale sequencing (GS) results, and to assess how these outcomes are associated with child and parent factors, including health literacy, numeracy, education, and medically underserved status. | Survey/ n = 1.763 | 1-item Perceived Understanding Scale – measured by a single Likert item (“How well do you understand your child’s test results?”).16-item Parent Personal Utility (PrU) Scale – subscales: Child Benefits, Affective Parent Benefits, Parent Control.12-item Parent FACToR Scale – subscales: Negative Emotions, Positive Feelings, Uncertainty, and Privacy Concerns.4-item Brief Health Literacy Tool (BRIEF) – assessing reading comprehension and confidence with medical materials.3-item Subjective Numeracy Scale (SNS) – capturing confidence and comfort with numerical information.Participants additionally classified as “medically underserved” based on language, income, insurance, residence, race, or ethnicity | Parents with higher health literacy demonstrated significantly greater perceived understanding of their child’s genomic test results (B = 0.03, p < .05) and fewer privacy concerns (B = −0.11, p < .001). Higher literacy was also associated with fewer negative emotions and greater emotional stability after result disclosure. In contrast, higher education and literacy were modestly associated with lower affective self/parent gains (B = −0.04, p < .05), suggesting that parents with higher literacy may process results more analytically and with less emotional reassurance. | Although direct data-sharing behaviors were not measured, the FACToR privacy concerns subscale served as an indirect indicator of attitudes toward data use. Parents with higher health literacy reported significantly fewer privacy concerns (B = −0.11, p < .001), indicating a greater implicit readiness to share genomic data under conditions of trust and comprehension. Conversely, parents meeting criteria for ‘underserved’ status (e.g., language barriers, race, or socioeconomic disadvantage) expressed higher privacy-related distress. |
| Lewis, C., et al.; 2020; UK | To explore young people’s understanding, motivations, concerns and decision-making regarding participation in the 100,000 Genomes Project, including their comprehension of genomic concepts, views on data sharing, and attitudes toward receiving secondary findings. | semi-structured interview/ n=27 people between the ages of 11 and 19; affected by rare diseases taking part in the 100, 000 Genomes Project | Five topics to enable an in-depth examination of young people's views:1. Knowledge and understanding of the term ‘genes and DNA’, ‘genomes’, ‘genome sequencing’2. Motivations for assenting/consenting to Genome Sequencing3. Concerns around Genome Sequencing4. Motivations and concerns regarding secondary findings5. Involvement in the decision-making process | Participants demonstrated a strong understanding of basic genetic concepts, accurately describing genes and DNA as determinants of personal traits and health, often using intuitive analogies such as an “instruction manual” or “fingerprint.” More complex terms like “genome” and “genome sequencing” was limited, especially among younger participants. Understanding improved with age and educational exposure, suggesting that foundational genetics knowledge is widespread, but genomic literacy remains partial and dependent on schooling and research participation.Many recognized heredity and the role of genetic faults in disease. A smaller subset showed more advanced knowledge, referencing inheritance patterns and gene–environment interactions. | Only a few were uncertain about how such data could potentially be misused. Concerns about insurance discrimination were rare and mitigated by trust in existing data protection regulations. Most participants trusted that their genomic data were securely handled, expressing confidence in the NHS and reassurance due to data deidentification. Several participants viewed data access by for-profit companies positively, recognizing their role in advancing research and drug development, while a few expressed ambivalence about commercial involvement.Decision-making was often shared with parents but older participants emphasized autonomy, describing participation as part of managing their own health. Involving young people in consent discussions was experienced as empowering (“made me feel important, not just a blood source”).Motivations for participation included desire for a diagnosis, understanding heritability, emotional closure, reassurance and altruistic reasons (helping others, contributing to science). |
| Chen, H.; 2021; China | To explore breast cancer patients’ perceptions of privacy within a hospital biobank context in China. | semi-structured interviews/ n= 40 chinese breast cancer patients aged from 28 to 71 recruited from the One Hundred Thousand Breast Cancer Patients' Genetic Testing Database | no formal instrument for assessing genomic knowledge or literacySemi-structured interviews exploring patients’ experiences with: breast cancer diagnosis and treatment; knowledge of genetic testing; motivations for biobank participation; concerns about participation; perceptions of privacy | Health literacy was not directly measured. Patients’ perceptions of privacy and data sharing reflected functional and critical health literacy aspects, such as understanding and evaluating the implications of disclosing medical and genetic information. | 4 of 40 participants explicitly raised privacy concerns regarding biobank participation. Their main worries included disclosure of cancer diagnosis to employers (n=1) and personal information leakage (n=3), often linked to prior negative experiences with data misuse and discrimination. When prompted, most other patients acknowledged similar concerns, emphasizing that genetic test results must remain strictly confidential.Concerns about contact information disclosure were widespread. Many patients feared that sharing personal data could lead to scams, unsolicited marketing of fake cancer treatments and privacy violations through mobile and online technologies (reflecting a general lack of trust in digital systems and strangers). minority of participants: expressed indifference, viewing loss of privacy as inevitable in modern society.Most patients associated privacy with informational rather than physical privacy, focusing on the protection of contact details, disease history and genetic test results. |
| Suckiel, S. A., et al.; 2022; USA | To explore diverse patients’ attitudes, perceived utility, barriers, and communication preferences regarding the potential clinical use of polygenic risk scores (PRS), in order to generate patient-centered evidence to inform future implementation strategies. | semistructured interviews/ n=30 patients who are participants (aged 35-50 years) of the BioMe Biobank | The interview explored participants’ understanding, attitudes, and concerns related to genomic testing through open-ended questions | Perceived Benefits: Participants demonstrated varying levels of understanding regarding the purpose and personal value of participating in genomic research. Engagement and perceived benefit depended on individual motivation, prior health knowledge, and perceived relevance to one’s own situation.Concerns: Limited comprehension of complex genetic or medical concepts led to uncertainty, confusion, and at times emotional distress. Some participants misinterpreted probabilistic findings as deterministic outcomes, reflecting gaps in genomic literacy.Barriers: Language barriers, technical terminology, and lack of accessible information hindered comprehension of research procedures and data use. Participants emphasized the importance of plain, nontechnical language and materials available in their preferred language. | Perceived Benefits: Participants often recognized the collective value of sharing data for advancing research, improving prevention, and benefiting families or communities. Altruistic motives and trust in institutions fostered openness toward data sharing within research settings.Concerns: Privacy and data misuse were prominent worries, often exceeding concerns about participation itself. Participants were uncertain about who might access their data and for what purposes, but many remained willing to share within trusted frameworks.Barriers: Unequal access to research participation (related to cost, insurance, language, or race) limited opportunities for data contribution. Limited understanding of how shared data are used and protected further reduced confidence. |
| Spector-Bagdady, K., et al.; 2022; USA | To explore how patients and their oncology providers understand, experience, and justify participation in precision oncology research, focusing on motivations, perceived benefits, risks, and challenges of informed consent within genomic cancer studies. | semi-structured interviews/ out of n = 20 participants n = 10 are MiOtoSeq patients | Interview guide developed from prior literature and embedded in the MiOtoSeq precision medicine protocol.The interview explored:Motivations for participating in precision oncology researchPerceived risks and benefits of genomic sequencingUnderstanding of the consent and enrollment processExperiences with data use and privacy within research participation | Patients demonstrated limited genomic literacy, with many describing their prior knowledge as coming from media or popular culture, such as Jurassic Park or TV documentaries. Several admitted they had only a vague sense of genetics or the Human Genome Project. This limited understanding led to misconceptions, with some equating research participation with personal medical benefit: One patient explained that joining the study ‘might save my life,’ although they knew it was not a therapeutic trial. Patients expressed strong curiosity and optimism: precision medicine ‘the future’ and describing current treatments as ‘archaic.’ Altruistic motivations were central: many emphasized a desire to help others or protect their families, stating that participation was ‘a way to watch out for my children later on when I’m gone’ or that they wanted to ensure their children ‘don’t go through this.’ Participation was framed as both an act of hope and a contribution to science, rather than a fully informed decision. | Patients perceived few personal risks in sharing genomic or medical data, often dismissing potential privacy concerns. Many described participation as effortless and worthwhile: ‘it really doesn’t cause discomfort or take time, so why wouldn’t you do it if it helps others in the future?’ Several referred to data sharing as a moral duty or legacy, expressing that their contribution could ‘help my family first and then other people.’ Concerns about discrimination or misuse of genetic data were rarely raised: one participant even joked that at age 70 he no longer feared such risks. Potential secondary findings: patients described them as both ‘a shield and a sword,’ but ultimately viewed knowing as better than ignorance. Data sharing was driven by trust, altruism, and emotional meaning rather than a nuanced understanding of privacy, data governance, or long-term use of genomic information. |
| Noohi, F., et al.; 2023; USA | To explore participants’ experiences with receiving and disclosing positive MODY genetic test results to biological relatives, their navigation of the healthcare system before and after diagnosis, and their perceptions and suggestions regarding the Registry’s framework for supporting cascade genetic testing. | one-on-one semi-structured interviews/ n=20 Adult participants (≥18 years) with a confirmed genetic diagnosis of MODY | Semistructured interview guide with open-ended questions developed from prior literature to explore: participants’ experiences with MODY diagnosis communication with healthcare providers result disclosure to relatives | Health literacy was not directly measured Participants demonstrated high self-advocacy and self-directed learning: independently researched MODY online; read articles; educated themselves to compensate for clinicians’ lack of knowledge | Participants expressed great trust in the registry and viewed participation as an opportunity to contribute to research and gain clarity about their own health status. They emphasized the importance of transparent and continuous communication about research progress and results. Some suggested that the information shared should be more tailored to their specific MODY subtype, while others would like the opportunity to exchange ideas with other participants in the future. Despite participants' positive attitudes, family members frequently declined further testing or participation. |
| Sabatello, M., et al.; 2024; USA | To compare the views of key stakeholders—patients/community members, clinicians, and IRB members—on PRS research, return of PRS results, clinical translation, and barriers/facilitators to behavior change, with attention to equity and historically marginalized groupswith a particular focus on how social determinants and health literacy influence patients’ understanding of PRS results and data-sharing preferences. | Qualitative descriptive content analysis of online focus groups/ n=25 patients/community members of the eMERGE-IV study, as part of the National Human Genome Research Institute | Semi-structured focus group with topics of:return of resultscommunication preferencesethics/regulatory issuesbarriers/facilitators to behavior change | 80% of participants were interested in participating, motivated by personal, familial and community benefits as well as altruism and support for scientific progress Participation was seen as both self-serving and socially beneficialParticipants emphasized the need for trusted, accessible communication and ongoing education to understand and apply genomic information. Limited health literacy and structural barriers constrained behavioral change, highlighting the importance of relational trust, language-sensitive education and continuous support mechanisms | Patients expressed divided views on data sharing: while some favored a “team-based” approach in which clinicians receive results to enhance care coordination, others opposed automatic disclosure or storage in electronic health records, citing privacy concerns, mistrust, and a desire for personal control. Across participants, a strong sense of data ownership emerged—individuals wanted to decide how and with whom their information is shared. Trust in researchers and healthcare professionals was a prerequisite for willingness to share but was not assumed automatically. Although many recognized the collective value of data sharing for advancing science and community health, concerns about privacy, discrimination and lack of transparency constrained unconditional participation. |
| Ta Park, V., et al.; 2022; USA | Examine attitudes toward precision health, motivations/barriers to participate, and acceptability of texting for recruitment/education among underserved Vietnamese Americans. | survey and participated in one of 3 focus groups/ n=37 Vietnamese Americans classified by age 18-30, 31-59, and ≥60 years enrolled in the All of Us Research Programm | 7-item 5-point Likert survey on genetics/genetic testing knowledge, attitudes, and intentions (e.g., genetics affect health; benefits of genetic info; comfort discussing testing; discrimination risk; willingness for risk/pharmacogenomic testing; willingness to join genetics research)3-item 5-point Likert scale on acceptability of texting to support precision-health understanding, testing uptake, and concept comprehensionQualitative themes addressed perceptions of precision health (genetic predisposition, prevention, environmental factors), motivations for participation (representation, knowledge, trust, incentives) | 3.95 overall and 3.94 for genetics indicate high knowledge-attitude-behavior scores, with no significant differences across age groups (p > .05), showing uniformly positive attitudes toward genetics and genetic testing. | Participants emphasized the need for Vietnamese American representation in research and wanted to “have a voice” in precision health to ensure culturally relevant treatments. Additional motivators included a desire for knowledge, previous research experience, incentives, and trust in the credibility of the research institution or community organization. |
| Raj, M. et a.; 2022; USA | To evaluate public deliberation as a method for understanding the values, preferences, and reasoning of current and former cancer patients regarding the use and sharing of health information in precision oncology, with particular attention to transparency, ethics and data control. | Evaluative descriptive mixed-methods study using a public deliberation approach with mixed methods (qualitative and quantitative pre–post evaluation) conducted in two full-day deliberative sessions (session 1: n = 28; session 2: n = 33) to assess process, knowledge gain, and reasoning dimensions/ n=61 current or former cancer patients (≥21 years) enrolled in the All of Us Research Program | Health literacy was not directly measured. The deliberation process itself functioned as an educational and evaluative instrument designed to enhance participants’ understanding of data sharing and institutional data use. Three key dimensions1. process: focusing on the design and implementation of the deliberation2. information: addressing how participants applied and sought new information during discussions3. reasoning: examining how participants balanced perspectives and reached collective understandingPre– and post–deliberation assessments captured changes in participants’ knowledge, reasoning, and understanding of ethical and organizational aspects of data sharing. The deliberation process thereby functioned as a qualitative–quantitative intervention aligned with the concepts of functional and critical health literacy. | 77 % of participants reported changes in their understanding of health information sharing and 60 % reported changes in their opinions about itKnowledge increased significantly regarding data privacy laws (p < .001) and public health data collection (p = .003)92–94 % rated expert presentations, Q&A sessions, and discussions with other participants as very or extremely helpful | Participants demonstrated critical reasoning about data sharing, balancing individual and societal perspectives and noting digital access barriers. They discussed text, email, and portal options, highlighting exclusion risks and concerns about discrimination, transparency, and third-party use. Trust, transparency, and autonomy were key values, though gaps in health data literacy persisted. |
| Smit, A. K. et al.; 2022; Australia | To assess the acceptability and perceived usefulness of a genetic counselor phone call for communicating personalized polygenic risk information for melanoma prevention within the Melanoma Genomics Managing Your Risk study, and to explore participants’ understanding, satisfaction, and information needs across different levels of health literacy | Concurrent mixed-methods design embedded within a randomized controlled trial/  n = 509 adults aged 18–69 years from the general population participating in the Melanoma Genomics Managing Your Risk Study n=346 completed the post-intervention satisfaction survey n=20 participated in follow-up qualitative interviews  n=411 no prior diagnosis of non-melanoma skin cancer n=83 previous diagnosis n=63 unsure  Interview subsample: n=16 no prior diagnosis n=3 history of non-melanoma skin cancer n=1 unsure | Health literacy was assessed with the item: “How confident are you filling out medical forms by yourself?”  Health numeracy was measured with the item: “How easy or hard do you find it to understand information that has a lot of numbers and statistics?”   n=20 semi-structured interviews explored participants’ experiences and understanding of their genomic risk results and the counseling process, providing qualitative insights into genomic literacy and interpretive understanding | 87.1% of participants reported being extremely or quite a bit confident in filling out medical forms; 12.9% indicated lower confidence 80.3% found it very easy or easy to understand numerical information; 19.7% reported difficulty Interview subsample (n = 20): 90% showed high confidence in both health literacy and numeracy | Out of 448 recorded participant questions, only 3 concerned what would happen to their saliva or DNA sample after the study, indicating minimal awareness or concern about data storage and secondary use. |
| Stallings, S. C., et al.; 2023; USA | To explore patient-level understanding of precision medicine, genetic literacy, numeracy and trust in healthcare providers among adults in a federally qualified community health center. | Mixed-methods cross-sectional study/ n = 26 adults aged 40–79 years; recruited from a community health center serving medically underserved populations Participants included cancer patients, individuals with a family member affected by cancer and caregivers of cancer patients | pre-focus group survey: 5-point, 3-item Subjective Numeracy Scale (SNS-3; Kripalani et al., 2019; McNaughton et al., 2015) 4-item Brief Health Literacy Screening Tool (Chew et al., 2004; Haun et al., 2012; Wallston et al., 2014) higher summed scores indicated greater literacy and numeracy.  Data were collected through four focus groups that explored participants’ understanding of genetics and precision medicine, their concerns and expectations regarding its use and their views on how information about precision medicine should be communicated to the public | Mean health literacy was 16.2 (Brief Health Literacy Screen) Mean numeracy was 9.4 (SNS-3)  85% felt confident completing medical forms  77% recognized the term “DNA”; 25% “genomics”; 12% “biobank”; 65% “hereditary”; 65% “chromosome”; 58% “genetic testing” The following factors were ranked by participants according to their importance for genetic testing decisions: 85% "insurance coverage"; 80% "receiving test results"; 77% "family impact"; 73% "privacy" and "counseling"; 72% "cost" and "trust in healthcare professionals"  Several participants expressed uncertainty about the potential risks and side effects of genetic testing, emphasizing a reluctance to feel like “guinea pigs” in untested medical procedures. At the same time, others highlighted the potential benefits through personal experiences: using genetic testing to confirm compatibility for a successful kidney donation within the family.  Participants highlighted multiple trusted sources and formats for learning about precision medicine; recommendations from healthcare professionals; churches; family members; patient testimonials; educational materials; community forums.  Trust, cultural relevance, and spiritual context were seen as key to improving understanding and engagement with genomic information. | Sharing data was not the main focus, but the following aspects became apparent:  Participants identified several barriers influencing engagement in precision medicine: psychological stress related to knowing genetic risks; financial and time constraints; mistrust in the healthcare system; uncertainty about the value of precision medicine; privacy concerns regarding data use; broader social and environmental challenges.  Participants described several perceived benefits in precision medicine: personal health insights; opportunities for disease prevention and improved treatment; contributing to the greater good through advancing science and helping others. |
